# Liquid Carbonic Snow

**Published:** 1910-11

**Authors:** William S. Ehrich

**Affiliations:** Evansville, Ind.


### Liquid Carbonic Snow.

BY WILLIAM S. EHRICH, M. D., EVANSVILLE, IND.

To Dr. Pusey, of Chicago, belongs the credit of having first used liquid carbonic snow in dermatology and having found a substitute for liquid air, which had been used experimentally on certain skin lesions.

Liquid air, on account of its unstable character and difficulty of preservation and handling, along with its great cost, was found to be utterly impractical for general use.

Then followed ethyl and methyl chlorides. They being more or less successful, further effort was made to find a substance which would have the chief characteristic of liquid air, its intense coldness.

Carbon dioxide snow is very easily obtained and in contradistinction to liquid air, the tanks of gas can be kept indefinitely. It is reasonable in price and can be easily handled.

The method of making the snow is simple, the principle being condensation of the gas on a suitable surface. After being made, it is gathered and moulded or shaved into suitable shape and size. Care must be taken by the operator to protect himself lest frost-bitten fingers result.

The snow is applied directly to the surface to be treated, some pressure being used. In growths larger than the thumb nail, the writer would prefer treating several small areas, leaving untounched portions between.

After the exposure, the area treated is depressed, white and solid, but in a short while it thaws out and becomes elevated and after several hours is covered with a vesicle containing clear aseptic serum.

The length of time required varies with the age of the patient and the location of the lesion, no hard and fast rules can be laid down. It averages between ten and forty seconds.

Another method of applying the snow is to attach an apparatus to the tank and apply the gas directly. The writer has never used this, as he considers it too dangerous, since absolute control can not be had of the destructive action.

As to the use of the snow, like the X-Ray, we are prone to consider it a cure-all, but, since it is of so recent advent, there has hardly been enough time for the exact status of this valuable agent to be established.

Among the diseases in which its use has been commended are vascular and pigmented naevi, lupus erythematosus and vulgaris, tattoo and gunpowder marks, chloasma, lichen planus and ruber and epithelioma.

Dr. Heidingsfeld has shown in two articles the final results in treating lupus erythematosus. These he does not consider encouraging as to permanent cure. All these cases, however, show temporary improvement. The same holds good for lichen ruber.

Since experiment has shown that the exposure of various micro-organisms to the snow is only inhibitory to their growth and not lethal, I am inclined to think that this is the reason for the temporary good results in lupus.

Much has been written recently about the use in epithelioma. The writer thinks, that this will be a very useful means of treating epithelioma before they have started to spread widely and deeply—the so-called benign variety—that are sometimes amenable to pastes other palliative treatments. However, in the malignant types the writer believes that the snow would be of advantage in relieving the pain, hemorrhage, etc., and checking the spread.

In powder marks, especially where the penetration is only superficial, when the vesicle ruptures many of the grains are extruded with the serum. I have never seen it used in tattoo marks but am sure of its usefulness in this field even though it may have to be pushed to destructive action.

In chloasma the slightest action is necessary, merely enough to cause desquamation.

The best results are obtainel in the treatment of naevi, both pigmented and vascular. The principle upon which the snow acts in the vascular is by causing an inflammation within the vessel—an endoarteritis—which blocks the vessel and thereby destroys the mark. In the pigmented by causing a vesicle which elevates the rete Malpighii in which is contained the pigment cells.

The most satisfactory case treated by the writer was in a six-weeks-old baby who had a bright red naevus in the center of her forehead; two applications of fifteen seconds duration served to entirely obliterate the lesion with apparently no scar; there is, however, a pink surface where the blister was situated which like that of any other blister will whiten in time.

In the adult the results are not so satisfactory as with children. In one case of a very large naevus extending from the bridge of the nose to one inch beyond the hair line on the scalp, the result was satisfactory in so much that the color was changed from a red to a light pink, but it probably will never be perfectly white. In this case, however, before he came under my observation every thing had been used on the lesion from the sun-glass and tattooing with nitric acid to Christian Science and, therefore, it was in poor condition to be worked on.

In conclusion I would say:

That carbon dioxide snow bids fair to become a very useful therapeutic agent.

That it is of value in the treatment of lupus and epithelioma.

That its use in treating naevi is par excellent.

That it is not without danger both to the patient and the operator.—*American Practitioner and News.*

